# Mesenchymal Stem Cells in the Treatment of Human Spinal Cord Injury: The Effect on Individual Values of pNF-H, GFAP, S100 Proteins and Selected Growth Factors, Cytokines and Chemokines

**DOI:** 10.3390/cimb44020040

**Published:** 2022-01-24

**Authors:** Lucia Slovinska, Denisa Harvanova, Jana Janockova, Jana Matejova, Peter Cibur, Marko Moravek, Timea Spakova, Jan Rosocha

**Affiliations:** 1 Associated Tissue Bank, Faculty of Medicine of P. J. Safarik University and L. Pasteur University Hospital, Tr. SNP 1, 040 11 Kosice, Slovakia; denisa.harvanova@upjs.sk (D.H.); jana.janockova@upjs.sk (J.J.); jana.matejova@upjs.sk (J.M.); marko.moravek@student.upjs.sk (M.M.); timea.spakova@upjs.sk (T.S.); jan.rosocha@upjs.sk (J.R.); 2 Department of Trauma Surgery, Faculty of Medicine of P. J. Safarik University and L. Pasteur University Hospital, Rastislavova 43, 041 90 Kosice, Slovakia; peter.cibur@upjs.sk

**Keywords:** spinal cord injury, mesenchymal stem cells, cell therapy, cytokines, neuromarkers

## Abstract

At present, there is no effective way to treat the consequences of spinal cord injury (SCI). SCI leads to the death of neural and glial cells and widespread neuroinflammation with persisting for several weeks after the injury. Mesenchymal stem cells (MSCs) therapy is one of the most promising approaches in the treatment of this injury. The aim of this study was to characterize the expression profile of multiple cytokines, chemokines, growth factors, and so-called neuromarkers in the serum of an SCI patient treated with autologous bone marrow-derived MSCs (BM-MSCs). SCI resulted in a significant increase in the levels of neuromarkers and proteins involved in the inflammatory process. BM-MSCs administration resulted in significant changes in the levels of neuromarkers (S100, GFAP, and pNF-H) as well as changes in the expression of proteins and growth factors involved in the inflammatory response following SCI in the serum of a patient with traumatic SCI. Our preliminary results encouraged that BM-MSCs with their neuroprotective and immunomodulatory effects could affect the repair process after injury.

## 1. Introduction

Spinal cord injury (SCI) is a devastating process leading to permanent neurological deficits for which there is currently no effective treatment. Injury, which comprises primary and secondary phases, provokes a cascade of events, that includes disruption of axons and blood vessels, primarily the damage to the blood–spinal cord barrier, extensive necrotic cell death, axon degeneration, demyelination, and the beginning of an immune response [1]. These destructive processes result in an incurable failure of the sensory, motor, and autonomic functions under the site of injury due to the limited regenerative capacity of the central nervous system (CNS). Usually, SCI starts with an accidental primary mechanical injury. Most SCI cases in humans of primary injury are impact/vertebral dislocations with persistent compression of the spinal cord. The majority of patients are left paralyzed with limited daily activities, which have serious psychosocial effects that are exhausting for patients and their immediate surroundings [2]. The secondary injury is the most crucial phase in the pathophysiological process of SCI concerns, where complex biochemical changes occur. Secondary changes include disturbance of local ionic concentrations, inflammation, vascular dysfunction through the spinal cord, edema, ischemia, cell apoptosis, excitotoxicity, and demyelination process, accumulation of inhibitory molecules, insufficient trophic factor support, and glial scar formation with a dual role in the pathological process of SCI, both protective and inhibitory [3]. All these processes together impair the regeneration process and lead to further death of neural and glial cells, widespread neuroinflammation, and persists for several weeks after injury [4,5].

Cell transplantation therapies are one of the promising strategies for the treatment of SCI. Different types of cells are the focus of attention and research for example neural stem cells [6,7,8], Schwann cells [9,10], olfactory ensheathing cells [11], glial precursor cells [12,13], based on their potential to eliminate the consequences of SCI, to replace lost neural tissue at SCI sites, such as support and promote axonal growth, improve myelination and bridge the wound site [14]. The most studied cell types used in SCI research are mesenchymal stem cells (MSCs). The International Society for Cellular Therapy (ISCT) defines these cells as being able to adhere to plastic culture dishes, with specific surface antigen expression (positive for CD73, CD90, CD105, negative for CD45, CD34, CD14 or CD11b, CD79 alpha or CD19, and HLA-DR) and with capacity for trilineage mesenchymal differentiation in vitro [15]. MSCs are easy to obtain and proliferate, with minimal ethical or immunological issues. MSCs can be isolated from human bone marrow (BM) from the superior iliac crest of the pelvis [16]. Other resources from which MSCs can be isolated are adipose tissue [17], umbilical cord Wharton’s Jelly [18,19], human placenta tissue [20], amniotic, chorionic membranes, umbilical cord, and placental decidua [21,22].

Inflammation is a key goal of experimental and clinical treatment. MSCs play an important role in this process because it was found that MSCs can suppress inflammation [23,24], down-regulate the microglia activity [25], minimize secondary injury, and protect the neural elements that survived the initial mechanical insult. Additionally, MSCs have been shown to differentiate into neural stem cells, neuron-like cells and stimulate neural stem cell proliferation to rebuild the damaged nerve tissue [26,27]. It has been reported that MSCs therapy after SCI leads to reduction of astrocytes proliferation and inhibition of glial scar formation [28].

MSCs perform their regeneration activity not only through the cell replacement of injured cells but also via strong paracrine impact. MSCs have been found to secrete a broad range of trophic factors, bioactive molecules with neuroprotective and neuroregulatory effects [29,30] to suppress immune response [31,32,33] and induce angiogenesis [34]. MSCs mediate direct neuroprotection by reducing neuronal sensitivity to glutamate receptor ligands and altering gene expression [28] Moreover, MSCs also express several interleukins, cytokines, and growth factors, such as interleukins IL-6 and IL-8, tissue inhibitor of metalloproteinase 2 (TIMP-2), monocyte chemoattractant protein-1 (MCP-1) and vascular endothelial growth factor (VEGF) detected by antibody array [35].

Predicting the long-term outcome after SCI and evaluating the effectiveness of cell therapy is an important component of the treatment strategy. Biomarkers that can easily be repeatedly measured within the blood or cerebrospinal fluid (CSF) of the patients to determine progressive neurological condition would be highly beneficial, as it would allow rapid determination as to whether the patient was improving, worsening, or showed sustained neurological stability in response to their current treatment, thus providing a biological surrogate outcome measure. The rupture of the blood–brain barrier (BBB) and either damage to the neurons and glia of the spinal cord tracts results in the release of biomarkers, largely cellular components, which are specific in the indication of nervous tissue damage and include neurofilaments (NF), S100 calcium-binding protein β (S100β) and glial fibrillary acidic protein (GFAP). GFAP, the main intermediary filament of astrocytes, is found only in the CNS and is upregulated in reactive gliosis following CNS injury [36]. GFAP is more astrocyte specific than S100 and the level of GFAP in the bloodstream increases as a result of rapid astrocyte destruction [37].

The members of the S100 protein family are multifunctional proteins involved in the regulation of a wide range of cellular processes. One of the members of this large family is the S100B calcium-binding protein physiologically mainly produced by astrocytes in the CNS [38] and its increased expression, as well as that of GFAP in blood and CSF represents a hallmark of astrocytic reaction to neural injury (reactive astrogliosis). Elevated levels of protein S100B originating from necrotic tissues might enhance or even amplify neurodegeneration by S100B-induced apoptosis. Measuring the level of S100B protein in serum and CSF had a potential value in the diagnosis of SCI in animal models [39,40]. These studies demonstrated that levels of S100B in serum and CSF significantly increased after experimental SCI in a time-dependent manner and therefore may be evaluated as a specific biomarker for acute SCI.

There are three major subunits of neurofilaments based on molecular weight, namely heavy (NF-H), medium (NF-M), and light (NF-L). A major structural component of large motor axons is the phosphorylated neurofilament subunit NF-H (pNF-H). It can be detected in significant amounts in CSF or serum as a result of neuronal loss after injury [41]. It is released from damaged and diseased neurons in significant amounts and may serve as a biomarker of neuronal damage in patients with amyotrophic lateral sclerosis [42], aneurysmal subarachnoid hemorrhage [43], and spinal cord injury [44]. Because neurofilaments are found only in neurons, the detection of released neurofilament subunits clearly reflects the level of damaged or destroyed neurons. The concentration of pNF-H should be useful for predicting the prognosis of each patient. 

**Figure 1:**
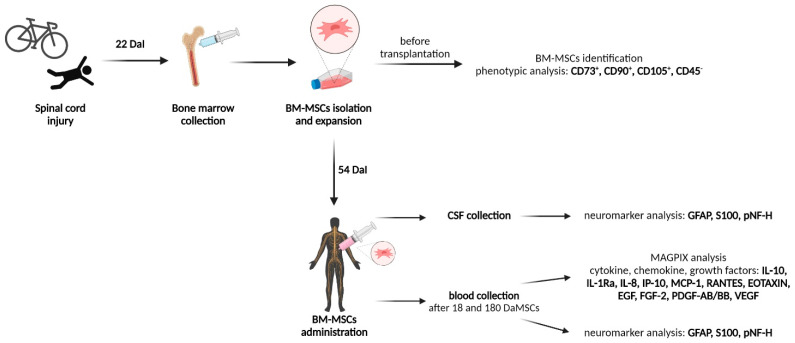
Schematic overview of the course of autologous bone marrow-derived mesenchymal stem cells (BM-MSCs) therapy in a patient with an injured spinal cord. DaI—Days after Injury, DaMSCs—Days after BM-MSCs transplantation. (Created with BioRender.com).

In our study, autologous bone marrow-derived MSCs (BM-MSCs) were isolated and expanded in vitro. After transplantation of BM-MSCs to a patient with SCI, the blood and CSF were analyzed for the presence of the pNF-H, S100 protein, and GFAP protein. Moreover, the dynamic changes to the chemokine/cytokine/growth factors’ profile in serum during the period of BM-MSCs treatment of a patient after SCI were examined (Figure 1). 

## 2. Results and Discussion

In our study, we have performed a descriptive analysis of the serum proteins’ level in the response to autologous BM-MSCs therapy of traumatic SCI. Cell transplantation treatment offers great potential to enhance the regeneration process after the injury. The blood and CSF of the BM-MSCs-treated patient were analyzed for the presence of the pNF-H, S100 protein, and GFAP protein. The chemokine/cytokine/growth factors’ profile was also observed in the serum during the follow-up period after the BM-MSCs treatment.

### 2.1. Patient

The patient was a 35-year-old male who had a bicycle accident. He has a complete transverse lesion at the level of thoracic 6 (T6), completely lost the sensation and movements below T6 with no voluntary anal contraction and sensation to deep anal pressure. During 1-year follow-up, we did not find any clinically adverse reaction related to the BM-MSCs transplantation.

### 2.2. BM-MSCs Cultivation and Administration

The majority of cell therapy trials use autologous cells, specifically stem cells, but MSCs are dominant in registered clinical trials. The limiting and decisive factors in the use of MSCs in cell therapy of human SCI in clinical practice are mainly the type of MSCs—the origin from which the cells are isolated, doses, cell delivery method, and precise timing of cell administration after injury [45,46]. 

Twenty-two days after the injury, the patient´s BM was collected in an amount of 25 mL. Subsequently, according to standard procedures, BM-MSCs were isolated and placed in 15 culture flasks with an area of 75 cm^2^. BM-MSCs were expanded under standard in vitro conditions. The cultivation itself lasted 32 days and finally, 54 days after the injury (DaI), the cells were ready for application in an amount of 4.08 × 10^6^/mL with 92.8% viability. In cell therapy trials the intrathecal cell delivery of MSCs predominates [45]. It was demonstrated that the injection of stem cells directly into the spinal cord is safe with minimal side effects. Moreover, intrathecal MSCs injection predominated over intravenous or intramedullary injection of stem cells in the clinical trials examining the use of stem cells in the treatment of SCI; whereas the time frame was 8 weeks after injury with the transplanted cell numbers ranging from 1 × 10^5^ to 40 × 10^7^ [47]. Although it was recognized, that any intervention in the treatment of SCI is effective at an early time point, within the first month after injury [14]. In our case, the autologous BM-MSCs were administered 54 DaI. As each organism is unique, also the cells derived from it behave with slight deviations from the standard. The slight prolongation of time from injury to cell application was due to the need to initiate the proliferation of autologous BM-MSCs and to achieve the required confluent cell layer during in vitro cultivation. Cell cultivation and the cultivation result itself can be influenced by several factors. Among other factors, the aging of MSCs can be a critical factor that affects the results of cell therapy. Transplantation of MSCs from young donors seems to provide better functional recovery through anti-inflammatory effects, vascular maturation, and neurogenesis potentially due to the dominance of trophic factor secretion [48]. Fortunately, the neurogenic differentiation capacity of MSCs is not correlated with donor age [49].

### 2.3. Phenotypic Analysis of BM-MSCs

**Figure 2:**
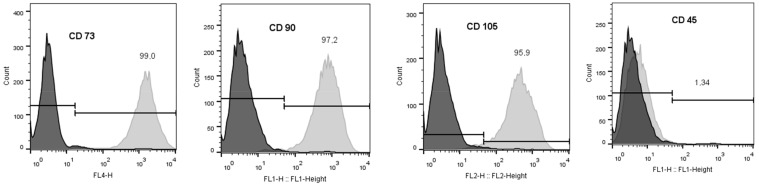
Surface markers expression in BM-MSCs. BM-MSCs were confirmed by flow cytometry analysis as positive for CD73 (99.00%), CD90 (97.2%), CD105 (95.9%), and negative for CD45 (1.34%).

Before application, MSC-specific markers were evaluated according to ISCT minimal definition criteria [15]. Cells did not express hematopoietic lineage marker CD45 (1.34%) and were positive for CD73 (99.0%), CD90 (97.2%), and CD105 (95.9%) thereby demonstrating a characteristic immunophenotype of human BM-MSCs (Figure 2).

### 2.4. The Levels of S100, GFAP and pNF-H Protein in the Serum and the CSF of a SCI Patient Treated with Autologous BM-MSCs

In contrast to a large number of reports on biomarkers in structural brain damage, only a few studies have investigated the role of biomarkers in patients with SCI. Quantitative evaluation of serum biomarkers could help to evaluate the course of damage and especially the success of tissue regeneration after the treatment. 

**Figure 3:**
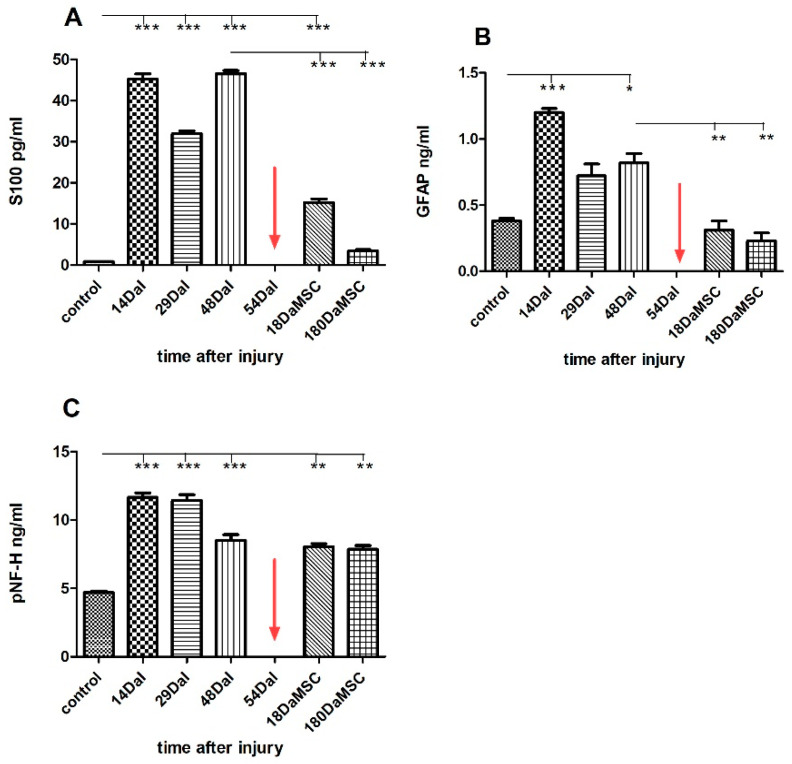
The level of S100 (**A**), GFAP (**B**)**,** and pNF-H (**C**) protein in the serum of an SCI patient treated with autologous BM-MSCs before and after the treatment. SCI caused a significant increase in S100, GFAP, and pNF-H protein levels in the patient’s serum in comparison with healthy controls (**A**–**C**). Administration of autologous BM-MSCs to the patient at day 54 post-injury resulted in a significant decrease in the levels of S100 and GFAP proteins in 18 days and 180 days follow-up period (**A**,**B**). The red arrow indicates the administration time of autologous BM-MSCs. Data are shown as the mean ± SD, * *p* < 0.05, ** *p* < 0.01, *** *p* < 0.001. DaI—Days after Injury, DaMSC—Days after BM-MSCs transplantation.

**Figure 4:**
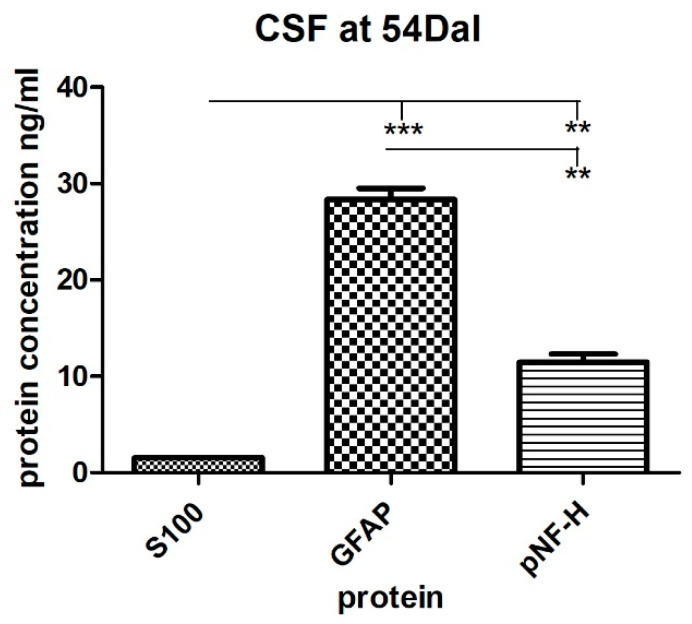
The levels of S100, GFAP, and pNF-H protein in the CSF of an SCI patient treated with autologous BM-MSCs. Protein values are obtained from CSF on the day of BM-MSCs application. The presence of glial cell proteins predominates over neuronal cell proteins. Data are shown as the mean ± SD, ** *p* < 0.01, *** *p* < 0.001. DaI—Days after Injury.

In the first two weeks after the spinal cord injury (14 DaI), high levels of S100, GFAP, and pNF-H proteins were detected in the serum. The serum levels of all three proteins were significantly (*p* < 0.001) higher than in the controls of healthy individuals (S100 = 45.22 ± 1.81 pg/mL vs. 0.81 ± 0.01 pg/mL, GFAP = 1.20 ± 0.04 ng/mL vs. 0.39 ± 0.03 ng/mL, pNF-H = 11.66 ± 0.47 ng/mL vs. 4,70 ± 0.14 ng/mL), with a tendency to fluctuate slightly over the next forty days (Figure 3). High levels of individual markers tended to persist for more than a month. Even six days before the application of the BM-MSCs, the protein levels maintained at significantly high values in comparison with controls of healthy individuals (S100 = 46.53 ± 1.09 pg/mL at 48 DaI vs. 0.81 ± 0.01 pg/mL in controls, GFAP = 0.82 ± 0.1 ng/mL at 48 DaI vs. 0.39 ± 0.03 ng/mL in controls, pNF-H = 8.51 ± 0.59 ng/mL at 48 DaI vs. 4.71 ± 0.14 ng/mL in controls). Generally, the detected high levels of S100, GFAP, and pNF-H proteins are directly related to the devastating consequences of the damage of the cells and spinal cord. High levels of pNF-H reflect progressive axonal loss due to secondary damage [50], whereas high levels of S100 and GFAP protein reveal glial cell degradation and rising blood levels of S100 protein mimic the severity of mechanical spinal cord injury [39,51]. Moreover, other studies determined that the pNF-H level in plasma was elevated in response to SCI and positively correlates with the severity of the SCI [52,53]. Elevated levels of GFAP were also reported in the serum of multiple sclerosis patients [54]. In our case, the autologous BM-MSCs were transplanted at day 54 DaI. A significant decrease in the serum level of mainly glial proteins, namely S100 (*p* < 0.001) and GFAP (*p* < 0.01) was detected after the transplantation at 18 DaMSC. The serum level of S100 protein decreased almost 3-fold (46.53 ± 1.09 pg/mL at 48 DaI vs. 15.18 ± 1.30 pg/mL at 18 DaMSC, *p* < 0.001) and the level of GFAP protein decreased two and a half times (0.81 ± 0.01 ng/mL at 48 DaI vs. 0.31 ± 0.09 ng/mL at 18 DaMSC, *p* < 0.01) in comparison with theirs levels before BM-MSCs application at 48 DaI. In the following 6 months after BM-MSCs transplantation, there was a further decrease in glial protein levels with a tendency to approach the values of healthy individuals. Regarding the pNF-H protein, there were no significant changes in its level after BM-MSCs application during the follow-up period. After the cell transplantation, there was a decrease in pNF-H protein level during the follow-up period, but not to the extent that we found in glial proteins. The prolonged increase in plasma pNF-H in patients with central nervous system disorders may be caused due to continuous axonal degeneration, such as Wallerian degeneration or secondary damage of axons [53]. The release of GFAP protein into CSF and the bloodstream with its increasing level in these fluids is a result of the mechanical disruption of the BBB integrity and of the disruption of the structural integrity of astrocytes at the same time. Furthermore, the major components of the axonal cytoskeleton—the neurofilaments—are released into CSF because of the axonal damage during neurodegeneration. Their levels in the CSF can be modified by the action of treatments commonly used in multiple sclerosis [55]. Therefore, the levels of S100 and GFAP protein in CSF of our patient was about 30-fold higher in comparison to the serum levels before BM-MSCs transplantation: 1559 ± 56.57 pg/mL in CSF vs. 46.53 ± 1.09 pg/mL in serum at 48 DaI for S100, 28.34 ± 1.63 ng/mL in CSF vs. 0.81 ± 0.01 ng/mL in serum at 48 DaI for GFAP (Figure 4). Detected glial protein levels in CSF were significantly higher than the pNF-H protein level. Because pNF-H is found only in axons, its detection in CSF, blood, or other body fluids indicates the release of this protein from axons. Cerebrospinal fluid levels of GFAP and pNF-H are elevated also in patients with spinal cord tethering [56], where levels of GFAP and pNF-H in CSF might reflect the ongoing scar formation and neuronal injury potentially responsible for progressive neurological deterioration. The concentration of pNF-H protein determined in the patient’s CSF was 11.44 ± 1.22 ng/mL and reflected the severity of spinal cord injury. The measured level of pNF-H protein in CSF was almost 10-fold higher when compared with the values obtained in a previous study [57]. In this study, the normal level of pNF-H in human CSF was set less than or equal to 0.94 ng/mL [57]. Our results are consistent with the finding, that the pNF-H levels in CSF from the patients with SCI were 5–10 times higher than the normal [44]. We evaluated dramatic changes in the levels of proteins, mainly of glial cells, when a significant decrease in S100 and GFAP protein levels in the serum were determined after the administration of autologous BM-MSCs at 18 DaMSC and 180 DaMSC, respectively. Therapeutic responses in the acute stage of SCI are not only due to intervention but also because of autorecovery (6–13%) [58]. However, in our case, in both glial proteins, there was a decrease of more than 50% in the serum levels after BM-MSCs treatment. At 6 months (180 DaMSC) after MSCs transplantation, both glial proteins maintained this trend, with approaching values that of healthy individuals. In addition to cell death, reactive astrogliosis occurs during secondary spinal cord injury, which is characterized by enhanced GFAP expression [59]. This apparent decline of both glial proteins in our study can be attributed to the response to the BM-MSCs-based therapeutic intervention. Application of BM-MSCs to the injured spinal cord could protect astrocytes from apoptosis and reduce GFAP overexpression. Human MSCs exert neuroprotection against stroke in vitro at least in part via an anti-apoptotic mechanism [60]. A similar finding was demonstrated by a previous study [61]. The authors suggested that paracrine factors secreted by MSCs promote astrocyte survival and are associated with GFAP downregulation after ischemic stroke in vitro via inhibition of two important mediators of the cellular response to external stimuli-p38 MAPK and JNK [61].

### 2.5. BM-MSCs Influenced Cytokine/Chemokine Levels in Injured Spinal Cord 

One of the key factors in secondary injury after SCI is inflammation. Therefore, subsequent successful treatment must focus on the suppression of this process and initiate repair events. The precise mechanism by which transplantation of BM-MSCs promotes functional recovery after SCI is still unclear. One of the options is that bioactive molecules secreted from the MSCs ameliorate functional deficits resulted from the damage [46]. Cytokines and growth factors with neuroprotective and regeneration-promoting effects belong to these molecules. To investigate the function of BM-MSCs in this process, we quantified the multiple inflammatory cytokines, chemokines, and growth factors in the patient´s serum using the multiplex techniques Magpix, where we can analyze one marker in the context of others in a wide dynamic range. 

**Figure 5:**
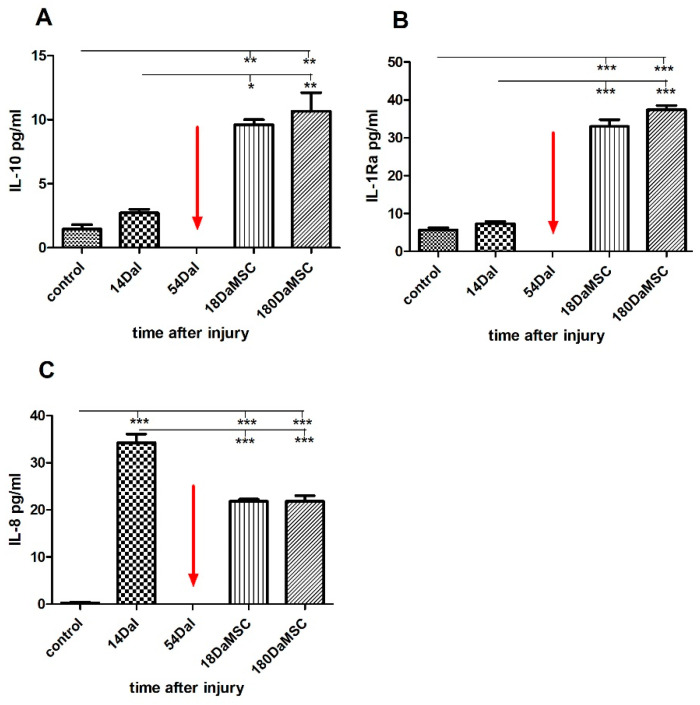
Influence of autologous BM-MSCs treatment on cytokine concentrations in the serum of an SCI patient. Levels of the cytokines IL-10 and IL-1Ra were significantly increased after BM-MSCs application at 18 DaMSC in comparison with their levels before the treatment (**A**,**B**). The level of IL-8 was significantly reduced at 18 DaMSC and 180 DaMSC due to BM-MSCs administration when compared with its level at 14 DaI before the treatment (**C**). The red arrow indicates the administration time of autologous BM-MSCs. Data are shown as the mean ± SD, * *p* < 0.05, ** *p* < 0.01, *** *p* < 0.001. DaI—Days after Injury, DaMSC—Days after BM-MSCs transplantation.

SCI caused an increase in interleukin IL-10 levels by almost 2-fold when compared to healthy controls (IL-10 = 2.72 ± 0.40 pg/mL at 14 DaI vs. controls = 1.45 ± 0.51 pg/mL, Figure 5A). IL-10 is a well-known anti-inflammatory cytokine. In addition to inhibiting the synthesis and release of pro-inflammatory mediators, it has been shown, that IL-10 exerts neuroprotective effects directly to neurons by suppressing cell death mechanisms due to the reduction of apoptosis. Furthermore, IL-10 plays a role in the activation of several signaling cascades involved in the survival and growth of spinal cord neurons in in vitro studies [62,63,64]. In vivo studies have been shown the positive effect of IL-10 in a reduction of secondary inflammatory [65]. IL-10 with IL-4 are preliminarily adopted as serologic markers to forecast SCI, and high serum levels of IL-4 and IL-10 may indicate a better prognosis [66]. We can see a similar response to injury in the expression of IL-1 receptor antagonist (IL-1Ra) protein, where protein levels within two weeks after injury slightly increased when compared to control levels (Figure 5B). IL-1Ra is an inhibitor of the pro-inflammatory effect of IL-1β. IL-1Ra with anti-inflammatory properties is secreted by various types of cells [67]. BM-MSCs treatment significantly increased the production of the anti-inflammatory cytokines IL-10 and IL-Ra at 18 DaMSC in comparison with the levels at 14 DaI before the treatment (IL-10 = 9.61 ± 0.52 pg/mL at 18 DaMSC vs. 2.72 ± 0.40 pg/mL at 14 DaI, *p* < 0.05, IL-1Ra = 33 ± 2.55 pg/mL at 18 DaMSC vs. 7.37 ± 0.79 pg/mL at 14 DaI, *p* < 0.001). Six months after BM-MSCs application, both cytokines retained significantly high serum levels with a tendency to increase over time when compared to control values. 

In contrast, interleukin IL-8 behaved in the opposite way to IL-10 and IL-1Ra cytokines (Figure 5C). IL-8 is a chemoattractant cytokine with a distinct target specificity for the neutrophil and is produced by a variety of tissues and blood cells [68]. IL-8 also supports cell homing and angiogenesis due to the expression of 41 core proteins in BM-MSCs through the PI3K/Akt pathway, which could promote the proliferation and migration of vascular endothelial cells [69]. In our study there was a significantly increase of IL-8 levels in the serum at 14 DaI when compared to controls of healthy individuals (IL-8 = 34.25 ± 2.62 pg/mL vs. controls = 0.3 ± 0.22 pg/mL, *p* < 0.001). The production of IL-8 was significantly decreased during the first two weeks after the BM-MSCs therapy at 18 DaMSC (*p* < 0.001) in comparison with its levels before the BM-MSCs application at 14 DaI. However, with increasing time after BM-MSCs administration, IL-8 did not tend to increase its expression in the serum of a BM-MSCs treated patient.

**Figure 6:**
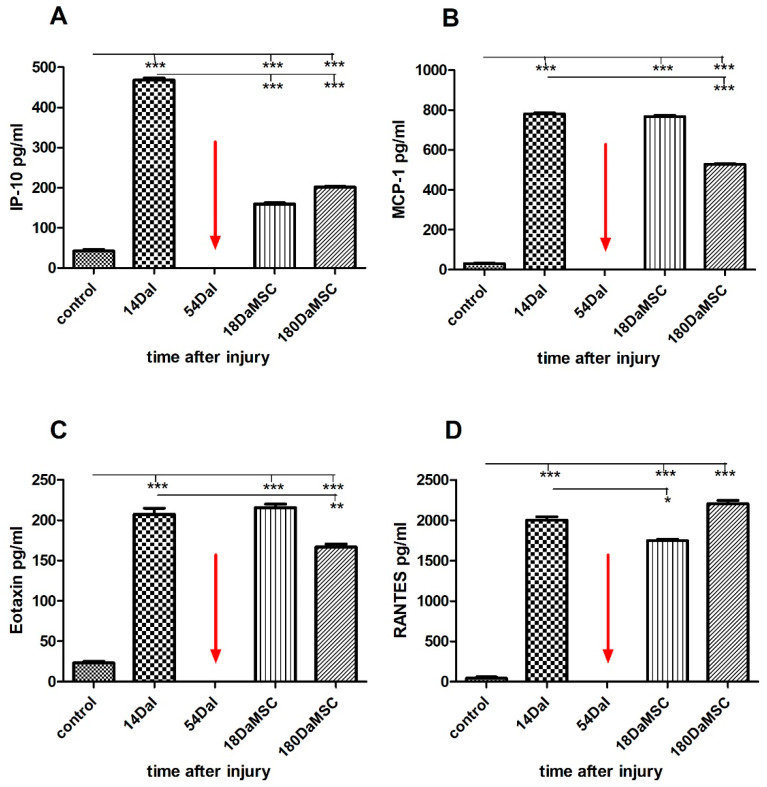
An overview of the level of the chemokine proteins in the serum of an SCI patient treated with autologous BM-MSCs. SCI caused a significant increase in IP-10 (**A**), MCP-1 (**B**), Eotaxin (**C**), and RANTES (**D**) protein levels in the patient’s serum at 14 DaI in comparison with controls. Administration of autologous BM-MSCs at day 54 post-injury resulted in a significant decrease in the levels of IP-10 and RANTES proteins at 18 DaMSC (**A**,**D**). The red arrow indicates the administration time of autologous BM-MSCs. Data are shown as the mean ± SD, * *p* < 0.05, ** *p* < 0.01, *** *p* < 0.001. DaI—Days after Injury, DaMSC—Days after BM-MSCs transplantation.

Similarly, as we observed in cytokines levels, the interferon-gamma inducible protein of 10 kDa (IP-10), monocyte chemoattractant protein-1 (MCP-1) and Eotaxin display significantly high expression during 14 DaI with 10–20 times higher levels when compared to control values (IP-10 = 468.10 ± 7.21 pg/mL at 14 DaI vs. controls = 45.25 ± 7.26 pg/mL, MCP-1 = 780.66 ± 8.40 pg/mL at 14 DaI vs. controls = 30.28 ± 5.03 pg/mL, Eotaxin = 207.43 ± 10.51 pg/mL at 14 DaI vs. controls = 23.17 ± 4.07 pg/mL, *p* < 0.001, Figure 6A–C). In the case of regulated upon activation, normal T cell expressed and presumably secreted (RANTES), there is an almost 50-fold significant increase in the serum level of this protein in comparison with the control of healthy individuals (RANTES = 2002.76 ± 55.92 pg/mL at 14 DaI vs. controls = 41.75 ± 20.58 pg/mL, *p* < 0.001) (Figure 6D). Administration of BM-MSCs caused a significant decrease (*p* < 0.001) in the level of IP-10 protein at 18 DaMSC in comparison with its level after injury at 14 DaI with a tendency to maintain a similarly low level within a six-month follow-up period after the cell administration. IP-10 is a small-inducible cytokine secreted during the inflammation by various cell types and acts as a potent inducer of apoptosis not only in infectious diseases but also during the development of the nervous system [70]. IP-10 is normally expressed in the spinal cord at low levels, but upon stimulation, the expression of this chemokine is significantly increased at glial cell accumulation sites [71]. IP-10 is also involved in neuronal injury and inflammation, where together with other glial chemokines, MCP-1 and RANTES, recruits leukocytes into inflammatory sites after CNS injury [72]. Interestingly, proteins IP-10 and IL-10 are interrelated; even in a previous study it was observed that IP-10 induces the production of IL-10 [73]. The significant decrease in the expression also occurred in the serum level of RANTES after BM-MSCs application at 18 DaMSC (*p* < 0.05) in comparison with its level after injury at 14 DaI. Based on our results, the RANTES protein tended to increase its level in response to BM-MSCs application. Similar results were obtained in other studies, where the expression of Ccl2/MCP-1 and Ccl5/RANTES was enhanced after MSCs transplantation and locomotor activity after spinal cord transection in mice was improved [74]. In the levels of the Eotaxin and MCP-1 proteins, we observed a similar pattern of changes in their serum levels after BM-MSCs application. Eotaxin is an eosinophil-selective chemokine, which recruits eosinophils during inflammatory conditions and plays a role in a variety of pathologic conditions [75]. Eotaxin and MCP-1 proteins maintained their high levels of 18 DaMSCs. However, after long-term follow-up, we observed a declining trend in the levels of these proteins. This finding can be explained by the fact that the chemokines RANTES and MCP-1, in addition to their chemotactic role, according to some published works, also play an important role in axonal regeneration and neuroprotection [76,77,78].

### 2.6. BM-MSCs Treatment Changed the Serum Levels of Growth Factors after SCI

In our study, we also monitored the changes in the expression of four growth factors in the serum (epidermal growth factor (EGF), vascular endothelial growth factor (VEGF), basic fibroblast growth factor (FGF-2), and platelet-derived growth factor (PDGF-AB/BB)) during the BM-MSCs treatment of SCI. 

**Figure 7:**
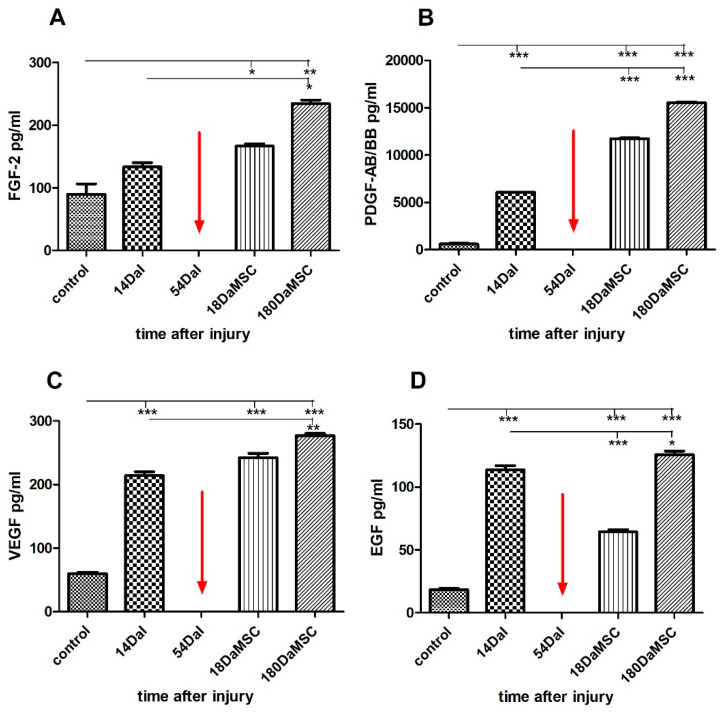
An overview of the level of the growth factors in the serum of an SCI patient treated with autologous BM-MSCs. Fourteen days after the injury, we recorded significantly higher levels of PDGF-AB/BB (**B**), VEGF (**C**), and EGF (**D**) in the patient’s serum when compared with control levels. FGF-2 protein levels also increased after SCI, but not significantly (**A**). BM-MSCs application showed a significant increase in FGF-2, PDGF-AB/BB, and VEGF levels. Conversely, there was a significant decrease in EGF protein expression after the application of autologous BM-MSCs at 18 DaMSC in comparison with its level before BM-MSCs treatment at 14 DaI (**D**). The red arrow indicates the administration time of autologous BM-MSCs. Data are shown as the mean ± SD, * *p* < 0.05, ** *p* < 0.01, *** *p* < 0.001. DaI—Days after Injury, DaMSC—Days after BM-MSCs transplantation.

Two weeks after the injury, we recorded significantly higher levels of PDGF-AB/BB, VEGF, and EGF in the patient’s serum compared with the control levels (*p* < 0.001) (Figure 7). Administration of autologous BM-MSCs caused a significant increase in levels of PDGF-AB/BB proteins at 18 DaMSC (*p* < 0.001). BM-MSCs application also caused an increase in FGF-2 and VEGF levels at 18 DaMSC, but not significantly. However, a significant increase in FGF-2 (*p* < 0.05) and VEGF (*p* < 0.01) occurred after a longer period after BM-MSCs application, at 180 DaMSC. VEGF is well-known as a specific mitogen promoting the growth and development of vascular endothelial cells. In combination with FGF-2 induces therapeutic angiogenesis [34]. Except, it is well established that VEGF is a potent angiogenic factor and that VEGF also plays a crucial role in the neurotrophic and neuroprotective activity in neurons, leading to increased axonal outgrowth in the nervous system. Furthermore, in vitro studies revealed that VEGF is a positive effector for growth cone movement and led to a considerable increase in dorsal root ganglia growth cone size [79,80,81]. VEGF may play an important role in neuroprotection as a therapeutic agent after acute traumatic SCI [82]. FGF-2 is widely known as an important player in wound healing for its proangiogenic effects. In addition to stimulating angiogenesis, FGF-2 improves neural cell growth and performs an important role in neurodegenerative diseases of the peripheral nervous system. In summary, its neurotrophic and angiogenic properties have a positive impact on the regeneration process [83]. As we can see from the measured values, the expression levels of FGF-2 and VEGF are interrelated. Not only does a combination of VEGF-A and FGF-2 have a strong synergistic effect on the new vessel formation in both in vivo and in vitro experimental conditions, even a combination of three growth factors FGF-2, VEGF-A, and PDGF-Ab/BB has a strong synergistic effect on the induction of neovascularization in experimental animal models in vivo [83]. Our results also reflect that all three factors have a similar course of the increase in the serum after administration of BM-MSCs. In contrast, the production of EGF protein was significantly reduced during the first 18 days after BM-MSCs application when compared with its level before BM-MSCs treatment at 14 DaI (*p* < 0.001). However, after 6 months follow-up, the production of EGF protein tended to increase. SCI causes glial cells adjacent to the lesion to significantly alter their phenotypes and activities. EGF seems to play a decisive role in this process by conversion of astrocytes to their reactive form [84]. MSCs themselves produce anti-inflammatory cytokines and growth factors, as well as stimulate their production in other cells, which thus also participate in the subsequent regeneration of damaged tissue. MSCs can improve astrogliosis by inhibition of the EGF receptor and ultimately upgrade the regeneration after SCI [85].

In summary, in this study, we compared the levels of multiple cytokines, chemokines, growth factors, and so-called neuromarkers in the serum of an SCI patient before and after the treatment with autologous BM-MSCs. This study established two main points: (a) the effect of autologous BM-MSCs administration after SCI on the levels of cytokines/chemokines/growth factors (EGF, Eotaxin, FGF-2, IL-10, IL-1Ra, IL-8, IP-10, MCP-1, PDGF-AB/BB, RANTES, VEGF) in the serum of an SCI patient, (b) the ability to detect the presence and the level status of SCI neuromarkers (S100, GFAP, and pNF-H proteins) in the serum and cerebrospinal fluid of an SCI patient treated with autologous BM-MSCs.

Our preliminary results demonstrate that SCI, as well as subsequent BM-MSCs therapy, caused significant changes mainly in the levels of growth factors and neuromarkers detected in the serum. The most significant changes occurred in the serum levels of the neuromarkers GFAP, S100 as well as the growth factors FGF-2, PDGF-AB/BB, and VEGF when compared to the levels before and after the treatment.

In summary, these findings demonstrate that neurodegenerative processes after traumatic SCI can be quantified biochemically by measuring S100, GFAP, and pNF-H levels in the serum or CSF. Together with inflammatory cytokines, growth factors and other biomolecules could emerge as surrogate markers and secondary outcome measures for neurodegenerative processes as well as for the rapid determination as to whether the patient was improving, worsening, or showed sustained neurological stability in response to the current treatment. 

Despite these very promising results, we acknowledge that the suitability of selected markers for SCI and the efficacy of BM-MSCs therapy after SCI have to be confirmed on a large group of patients in randomized clinical trials.

## 3. Material and Methods

### 3.1. Patient Enrolment

A 35-year-old male with acute SCI and paraplegia after a bicycle accident, injured at the thoracic T6 level, was enrolled in our Department of Trauma Surgery. This study was approved by the institutional Ethics Committee of L. Pasteur University Hospital (06/EK/2020). Ethical guideline provisions from the Helsinki Declaration were followed. Written informed consent and the baseline status were obtained from all subjects before the study. 

### 3.2. Bone Marrow Collection and MSCs Isolation

The BM was collected in the operating room under the general narcosis. BM was aspirated aseptically from the iliac crest. BM aspirates were combined with a 5% (*v*/*v*) Heparin anticoagulant (Becton Dickinson, San Jose, CA, USA) in the syringe to prevent coagulation. Directly after BM aspiration, all tubes were gently mixed and immediately (<1 h) transported on ice to the Associated Tissue Bank, Faculty of Medicine of P. J. Safarik University and L. Pasteur University Hospital. All cell manufacturing procedures were performed under strict sterile conditions. In the laboratory, BM aspirates were directly processed for the MSCs extraction using the red blood cell lysis method. Erythrocyte lysis buffer (10× stock solution: ultrapure water with 1.5 M ammonium chloride (NH_4_Cl), 100 mM potassium bicarbonate (KHCO_3_), 1 mM ethylenediaminetetraacetic acid (EDTA) (all from Sigma-Aldrich, Merck KGaA, Darmstadt, Germany) was added to the total volume of BM in the proportion 1:4 (BM volume: 1 x lysis buffer). The conical centrifugal tube was mixed manually for 10 min on ice and centrifuged at 200× *g* for 10 min at 4 °C. Promptly, after centrifugation, the supernatant was discarded and the pellet was resuspended in Dulbecco’s Modified Eagle’s Medium (DMEM; Gibco; Thermo Fisher Scientific, Inc., Waltham, MA, USA) and washed twice using centrifugation in the same conditions (10 min at 200× *g*, 4 °C). Total cell number was determined by using a Bürker cell counting chamber. Finally, mononuclear cells were seeded at a density 1 × 10^6^ cells/cm^2^ in 75 cm^2^ ventilated tissue culture treated flasks and cultivated in the complete cultivation medium containing alpha-modified minimum essential medium (α-MEM) supplemented with 10% fetal bovine serum (FBS) and 1% (*v*/*v*) antibiotic/antimycotic solution (all from Gibco, Thermo Fisher Scientific, Inc., Waltham, MA, USA). After initial incubation for 2–3 days, the flasks were carefully washed to remove nonadherent cells and the medium was replaced with a fresh culture medium. The cultures were maintained in a humidified incubator at 37 °C with 5% CO_2_ and the medium was changed twice a week. The pathogen- and virus-free status was confirmed according to the safety analysis such as sterility, mycoplasma, and endotoxin detection before transplantation. In addition, tri-lineage differentiation tests in vitro and phenotypic control of BM-MSCs at 0 passages were used in this clinical study. The final product was prepared as 4.08 × 10^6^ cell/dose of autologous BM-MSCs with 92.8% of viability. The cell viability was evaluated with trypan blue staining. 

### 3.3. Phenotypic Analysis of BM-MSCs before Application

Before application, immunophenotypic analysis of BM-MSCs was performed with a human MSC phenotyping kit (Miltenyi Biotec, Bergisch Gladbach, Germany). A minimum of 2 × 10^5^ cells were resuspended in PBS (Sigma-Aldrich, Merck KGaA, Darmstadt, Germany) with 2% FBS (FBS, Thermo Fisher Scientific, Inc., Waltham, MA, USA), centrifuged at 300× *g* for 10 min, stained with a cocktail of antibodies CD 45 FITC, CD 90 FITC, CD 105 PE, CD 73 APC for 10 min in dark, washed with PBS containing 2% FBS and centrifuged at 300× *g* for 10 min. Cells were analyzed with a Becton Dickinson FACSCalibur using CellQuestPro software (Becton Dickinson, San Jose, CA, USA).

### 3.4. Preparation of BM-MSCs for Clinical Application

Two weeks before application, the FBS was changed to the patient’s autologous serum during cultivation. After reaching a confluence of above 90% (4–5 weeks from initial seeding), cells from passage 0 were washed twice with sterile PBS and treated with 0.25% Trypsin–EDTA solution (Gibco; Thermo Fisher Scientific, Inc., Waltham, MA, USA) for 2 min at 37 °C. Cells solution was collected and centrifuged at 200× *g* for 10 min at 4 °C. The total number of cells was counted after discarding the supernatant. Before application, the cell pellet was resuspended in sterile 0.9% saline solution in a final volume of 1 mL. For transportation, BM-MSCs in sterile normal saline were maintained in a temperature-controlled environment of approximately 4 °C under sterile and dark conditions. The stem cells suspension was transported as rapidly as possible. 

### 3.5. BM-MSCs Administration

The transplantation of BM-MSCs (4.08 × 10^6^/mL) was performed by intrathecal injection in the operating theater under general anesthesia. The surgical wound was closed in a regular manner. The transplantation was performed using freshly harvested cells on 54 DaI.

### 3.6. Sample Collection

#### 3.6.1. Serum

**Figure 8:**
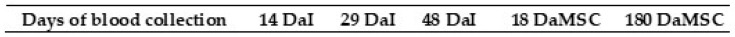
Time points of blood collection. Note: DaI—Days after Injury; DaMSC—Days after BM-MSC transplantation.

Whole blood was collected in a covered test tube without anticoagulants and the clot was then removed by centrifuging at 1500× *g* for 10 min. Serum samples were collected at given time points (Figure 8). Controls were presented by healthy individuals of the same age and gender as the patient (*n* = 3). After centrifugation, human serum was aliquoted and stored at −80 °C until detection. 

#### 3.6.2. Cerebrospinal Fluid

To minimize the number of invasive procedures to the injured patient, CSF was obtained during the same procedure as the BM-MSCs were administered intrathecally on 54 DaI, in the quantity of 5 mL. Collected CSF was separated by centrifugation at 300× *g* for 10 min to remove any cells or large cellular fragments, aliquoted, and stored at −80 °C until detection.

### 3.7. Multiplex Assay for Analysis of Cytokine/Chemokine/Growth Factors

Concentrations of 11 analytes (EGF, FGF-2, IL-10, IL-8, IL-1Ra, IP-10, MCP-1, Eotaxin, PDGF-AB/BB, RANTES, VEGF) were quantified by a triplicate for each serum sample/time point using MILLIPLEX^®^ Assays (Millipore, Merck KGaA, Darmstadt, Germany) to determine the mean of fluorescence intensity (MFI) values according to the manufacturer’s protocol and the MAGPIX Luminex platform. xPONENT software version 4.2 for MAGPIX (Luminex Corporation, Austin, TX, USA) and Bio-Plex Manager 6.1 (Bio-Rad Laboratories, Hercules, CA, USA) were used for data analysis. After creating a standard curve, concentrations were interpolated for each sample and expressed as pg/mL.

### 3.8. ELISA Measurement of Human S100, GFAP, and pNF-H 

The concentrations of target proteins in the serum and the CSF of the patient were detected using the Human S100, GFAP, and Phosphorylated Neurofilament H (pNF-H) Kits (all from Millipore, Merck KGaA, Darmstadt, Germany), following the manufacturer’s protocols. Samples were added into the wells of a polystyrene plate with pre-coated monoclonal antibodies against the specific protein of our interest and washed out with wash buffer after their respective additions to the wells. TMB substrate was used for coloration after the enzyme conjugate had already been thoroughly washed out of the wells. TMB reacted to form a blue product from the peroxidase activity and finally turned yellow after the addition of the stop solution. The optical density value of the target analyte in the sample was assessed at 450 nm using a microplate reader (TriStar LB941, Berthold Technologies, Bad Wildbad, Germany). Then, the standard curve was generated and the protein levels of tested samples were calculated according to the manufacturer’s guidance.

### 3.9. Statistical Analysis

Statistical differences among the groups were assessed by one-way ANOVA and Dunn’s multiple comparison test as post hoc analysis using GraphPad Prism software (GraphPad Prism Software, Inc.). Values *p* < 0.05 were considered statistically significant (* *p*-value of < 0.05, ** *p*-value of < 0.01, *** *p*-value of < 0.001). The concentrations of proteins were expressed as the mean ± standard deviation (SD).

## Data Availability

The study did not report any data.
